# Models by J. Lee, M. D., D. D. S.

**Published:** 1858-11

**Authors:** J. Lee


Models, Lee's method of obtaining:—

I take the impression in the usual manner, preferring simply the clean yellow wax to any preparation of it. I fill the mould with prepared plaister, raising above the teeth and gums (with sheet iron ring) a body about half an inch high. This I trim smooth before hardening, melt off the wax and dry the plaister cast well in the fire, wrap it in two or three turns of common stiff paper, pass a string round it, and you have a cup prepared to receive the metal.

This consists of one part by weight of type metal, and two parts of lead, previously melted together and run into ingots, to make a perfect alloy. This alloy is easily fused, and retains its fluidity at a low heat; it is hard when cold, and in cooling does not shrink, the antimony in the type metal having the property of expanding in cooling.

This alloy melted, but not quite hot enough to show the blue color, the proper degree of heat can be easily tested by a strip of paper; if the paper is not charred, but merely scorched, the temperature is right. It is now poured on the plaister model so as to cover the gums, and nearly to the top of the teeth; the teeth should not be covered the first pouring, as there is generally an escape of vapor from the plaister, even after well drying; as soon as this ceases, having kept the metal hot, the mould should be filled up forming a solid body above the teeth. When cold take out the plaister, envelope the metal cast just made in paper, and make the reverse swage by pouring in the metal somewhat colder than first used.

I separate the casts and taking the first or female die, fill up the impression of the teeth with bits of soft paper, and make by pouring as above, an impression of the gums, &c., without the teeth. This I use in giving my first fit to the gold plate, taking now the swage with teeth, I apply the gold plate and cut away as much gold as will enable me with but little force, to drive the dies together; part them, trim the plate as may be necessary, re-apply, and with a sledge hammer drive up by striking round the edges and finishing by a few blows in the middle. You will find your plate will fit the mouth with accuracy.

Yours,

J. LEE, M. D., D. D. S.

